# The Action of Microbial Products on Microbes and on the Organism

**Published:** 1890-11

**Authors:** 


### The Action of Microbial Products on Microbes and the on the Organism.

"Microbes are always the indispensable cause of virulence; they are always the cause of immunity, I dare not say the indispensable cause, but they only produce their effects by means of the chemical matters that they secrete." With these words Prof. Bouchard premises, in the *Revue de Medecine* for July, one of the most comprehensive studies of the action of the products secreted by pathogenic micro-organisms that have appeared. The subject has been studied experimentally by the action of bacterial products on microbes; by the action, both harmful and useful, of these products on animal organism; by the action that the products of a microbe exercise on the infection produced not only by that, but also by another microbe, and by an examination of the measures by which these products influence infection, by their action both on the microbe-destroying state of the humors and on phagocytosis.

The products of the vitality of a microbe, as of all living cells, are multiple. Many of these substances are not toxic, but the toxic matters of a single kind of microbe are numerous; they are diastases, alkaloids, volatile acids, etc. And the author believes that the inoculable are distinct from the toxic matters. Among the local lesions of infection, the chemical alterations of tissue depend on diastase, but it is extremely probable that the paralysis of the leucocytes, the obstacle to phagocytosis, is due to some other toxic substances, called toxines. Infectious fever seems to be due to diastatic substances, and perhaps to certain cellular alterations that occur in the liver, kidneys, and muscles, while any nervous phenomena depend on the toxines. Whatever the substance that produces immunity, it is not believed that it is a diastase.

The conclusions deduced from the experiments seem to prove that among the substances secreted by microbes is a substance capable of injuring directly the development, multiplication, and secretion of the micro-organism, although this is indirectly favorable to the microbe by chemically modifying the environment.

There are substances secreted by a microbe that are either inhibitory or favorable for microbes of other species. There are microbes that secrete substances poisonous to animals, and it is this toxicity that constitutes the virulence of a microbe.

While there are pathogenic microbes that secrete inoculable matter, it is not by its presence alone that this matter produces immunity, for in some way the inoculable matters so impress the animal organism that even when they are eliminated the humors permanently remain less propitious to the vitality of the same microbe. The inoculable substances change the activity of the cells in some fashion, so that even when eliminated the leucocytes, though confronted by the same microbe, more abundantly effect diapedesis and more energetically accomplish their phagocytic function.

Though the soluble matters of a microbe when injected at the same time with an inoculation of the same microbe render the infection more intense, yet the same matters injected some days before inoculation, far from aggravating the infection, inhibit or attenuate it. With antagonistic microbes—that is to say, those in which a simultaneous inoculation generally develops one only —it is noticed that the soluble matters of the stronger inhibit the weaker, though if injected at the same time with an inoculation of the weaker, they produce a moderation and attenuation of the infection most pronounced if given in the same locality. Auxiliary microbes may, by the inoculation of one or the injection of its soluble products, allow the other to develop in an animal that is naturally refractory, though in case the virulence of the microbe should be slowly attenuated, it would only develop in an unrefractory animal.

The bacteria-destroying condition of the animal organism produced by the injection of bacterial matters should appear at the end of the first twenty-four hours; and it is neither suppressed nor suspended by a new injection of such substances as have conferred the immunity. In animals that have a natural or acquired immunity, and that are capable of resisting a pathogenic microbe by phagocytosis, the soluble products of that microbe would inhibit phagocytosis, while in animals having no immutity, natural or acquired, but capable of resisting non-pathogenic or attenuated pathogenic microbes by phagocytosis, the products of a virulent microbe will inhibit the phagocytosis. These results prompt the question of what other substances, microbial or not, can produce the same effect on phagocytosis, or is the latter the mechanism by which they act?—*N. Y. Med. Jour.*

